# The Chloroplast Genome Sequence of *Scutellaria baicalensis* Provides Insight into Intraspecific and Interspecific Chloroplast Genome Diversity in *Scutellaria*

**DOI:** 10.3390/genes8090227

**Published:** 2017-09-13

**Authors:** Dan Jiang, Zhenyu Zhao, Teng Zhang, Wenhao Zhong, Chunsheng Liu, Qingjun Yuan, Luqi Huang

**Affiliations:** 1 School of Chinese Materia Medica, Beijing University of Chinese Medicine, Beijing 102488, China; jiangxiaodan1027@163.com; 2 State Key Laboratory Breeding Base of Dao-di Herbs, National Resource Center for Chinese Materia Medica, China Academy of Chinese Medical Sciences, Beijing 100700, China; zhaozhenyu9607@163.com (Z.Z.); zhangteng123589@163.com (T.Z.); zwh3141592@163.com (W.Z.)

**Keywords:** *Scutellaria baicalensis*, chloroplast genome, genomic resource, single nucleotide polymorphisms, microsatellites, indels

## Abstract

*Scutellaria baicalensis* Georgi (Lamiaceae) is the source of the well-known traditional Chinese medicine “HuangQin” (*Radix Scutellariae*). Natural sources of *S. baicalensis* are rapidly declining due to high market demand and overexploitation. Moreover, the commercial products of *Radix Scutellariae* have often been found to contain adulterants in recent years, which may give rise to issues regarding drug efficacy and safety. In this study, we developed valuable chloroplast molecular resources by comparing intraspecific and interspecific chloroplast genome. The *S. baicalensis* chloroplast genome is a circular molecule consisting of two single-copy regions separated by a pair of inverted repeats. Comparative analyses of three *Scutellaria* chloroplast genomes revealed six variable regions (*trnH-psbA*, *trnK-rps16*, *petN-psbM*, *trnT-trnL*, *petA-psbJ*, and *ycf1*) that could be used as DNA barcodes. There were 25 single nucleotide polymorphisms(SNPs) and 29 indels between the two *S. baicalensis* genotypes. All of the indels occurred within non-coding regions. Phylogenetic analysis suggested that Scutellarioideae is a sister taxon to Lamioideae. These resources could be used to explore the variation present in *Scutellaria* populations and for further evolutionary, phylogenetic, barcoding and genetic engineering studies, in addition to effective exploration and conservation of *S. baicalensis*.

## 1. Introduction

*Scutellaria baicalensis* Georgi (Huang-qin or Chinese skullcap), a perennial herb of the family Lamiaceae, is widely distributed in northern China and adjacent areas of Japan, Korea, Mongolia, and Russia. The root of this herb (*Radix Scutellariae*) is used as a traditional Chinese medicine, which was first recorded in Shennong’s Classic Materia Medica (Shen Nong Ben Cao Jing) in ca. 100 BC and is officially listed in the Pharmacopoeia of China. This medicine is widely used for clearing heat dampness and purging fire detoxification. Pharmacological data reported in the literature suggest that *Radix Scutellariae* shows beneficial effects in the treatment of hepatitis [1], tumors [2], cardiovascular diseases [3], and inflammatory diseases [4].

In recent decades, due to high market demand, overexploitation, habitat destruction and ecosystem deterioration, there has been a rapid decline in natural sources of *S. baicalensis*. Furthermore, this species has been listed as a class III conserved medicinal plant in China. Considering the harvest pressure on wild populations and the increasing demand for the root of *S. baicalensis*, many cultivation programs have been initiated in China since 1958 [5]. However, there has been a lack of scientific exploration of the plant, and the maintenance of genetic diversity through cultivation can reduce the incentive to conserve wild populations [6]. Therefore, it is important to compare the genetic diversity and population structure of *S. baicalensis*.

Because of the high price of *Radix Scutellariae*, its commercial products have often been found to contain adulterants (such as *Scutellaria. amoena*, *Scutellaria. rehderiana*, and *Scutellaria. viscidula*) in recent years. The contents of the biologically active compounds of these adulterants are distinct from those of Huang-qin. These inherent differences may cause a series of inconsistent therapeutic effects and quality control problems in the herbal medicine industry [7]. For effective exploration and conservation, accurate discrimination of *Radix Scutellariae* and its adulterants would be beneficial.

In *S. baicalensis*, molecular resources have been developed in recent years for both evaluation of its genetic diversity and species identification. For example, microsatellite simple sequence repeat (SSR) markers and chloroplast fragments (*trnL-psbF*, *atpB-rbcL*, *trnH-psbA*) have been used to evaluate population structure and genetic diversity patterns [8,9,10], and chloroplast genome markers (*rbcL*, *matK*, *trnH-psbA*) have been employed to discriminate *S. baicalensis* and its adulterants [7,11]. However, these markers are of low diversity and only provide a limited resolution in identifying closely related taxa. The development of more effective genetic resources will be necessary to foster efforts regarding the identification, conservation, utilization, and breeding of this species.

In the past decade, an increasing number of researchers have focused on the chloroplast genome. The chloroplast is a photosynthetic organelle that provides essential energy for plant cells [12]. The chloroplast genome structure is characterized by a small genome size, with a circular quadripartite structure ranging from 120–165 kb in length, containing a pair of inverted repeats (IRs) separated by a large single-copy region (LSC) and a small single-copy region (SSC) [13]. Moreover, genetic information from the chloroplast genome is inherited maternally, and the chloroplast genome is therefore a good indicator of maternal ancestry. Chloroplast genome sequences are known for their relatively stable genome structure, gene content, gene order and slow rate of mutation [14,15]. For these reasons, the chloroplast genome is a potentially useful tool for phylogenetic studies and species identification [16,17,18]. Most studies have shown that chloroplast genome mutations are not random but cluster at “hotspots” [16,19,20,21], and this mutational dynamic has resulted in highly variable regions in the genome. Chloroplast genome single nucleotide polymorphism (SNP), SSR, and indel markers have been developed in recent studies, as they show high abundance in the genome and improve population resolution [22,23,24,25].

In this study, we sequenced two *S. baicalensis* genotypes from different geographical locations and compared the resulting sequences with the published chloroplast genome of *Scutellaria insignis* and *Scutellaria lateriflora*. The first objective was to evaluate the interspecific variation in *Scutellaria* and retrieve valuable chloroplast molecular markers for species identification. The second objective was to retrieve valuable intraspecific variable chloroplast genome molecular markers, including SSRs, indels and SNPs, for *S. baicalensis*. The genomic and marker resources described in this paper will not only provide a molecular toolkit for species identification at species level, but also allow accurate genetic diversity and population structure of *S. baicalensis*.

## 2. Materials and Methods

### 2.1. Taxon Sampling, DNA Extraction and Sequencing

Fresh leaves of *S. baicalensis* from two individuals were collected from Huma, Heilongjiang Province (BOP028265), and Jiangxian, Shanxi Province (BOP028266). Fresh leaves from each accession were immediately dried with silica gel before DNA extraction. Voucher specimens were deposited in herbaria of Institute of Chinese Materia Medica (CMMI), China Academy of Chinese Medical Sciences. Total genomic DNA was extracted following a modified cetyltrimethyl ammonium bromide (CTAB) protocol [26] and purified using the Wizard DNA CleanUp System (Promega, Madison, WI, USA).

The chloroplast genomes of *S. baicalensis* plants were sequenced using the short-range polymerase chain reaction (PCR) method reported by Dong et al. [13]. The PCR protocol was as follows: preheating at 94 °C for 4 min, followed by 34 cycles at 94 °C for 30 s, annealing at 55 °C for 30 s and elongation at 72 °C for 1.5 min, with a final extension at 72 °C for 10 min. PCR amplification was performed in an Applied Biosystems VeritiTM 96-Well Thermal Cycler (Model#: 9902, made in Singapore, Applied Biosystems, Waltham, MA, USA).

The chloroplast data generated in this study was submitted to GenBank (accession number MF521632 and MF521633).

### 2.2. Chloroplast Genome Assembly and Annotation

The DNA sequences were manually confirmed and assembled using Sequencher v5.4 (Gene Codes, Ann Arbor, MI, USA) software. DNA regions showing poly-structures or difficulty in amplification with universal primers were further sequenced using newly designed primer pairs to confirm reliable, high-quality sequencing results. The four junctions between the IRs and SSC/LSC regions were checked via amplification with specific primers as described previously [13].

The whole chloroplast genome was annotated using DOGMA (Dual Organellar GenoMe Annotator) [27] to identify coding sequences, ribosomal RNA (rRNAs), and transfer RNAs (tRNAs) using the chloroplast/bacterial genetic code. BLASTX and BLASTN searches were performed to accurately annotate the genes encoding proteins and the locations of tRNAs. The whole chloroplast genome map was generated using GenomeVx [28].

### 2.3. Codon Usage

Codon usage was determined for all protein-coding genes. To examine the deviation in synonymous codon usage, avoiding the influence of the amino acid composition, the relative synonymous codon usage (RSCU) was determined using MEGA 6 software [29].

### 2.4. Analysis of Tandem Repeats and Microsatellites

The Perl script MISA (MIcroSAtellite) [30] was employed to search SSR loci in the chloroplast genome sequence, with the threshold value of the repeat number set as ≥10 for mononucleotide repeats, ≥5 for dinucleotide repeats, ≥4 for trinucleotide repeats, and ≥3 for tetranucleotide repeats, pentanucleotide repeats, or hexanucleotide repeats.

REPuter was used to visualize the repeat sequences in *S. baicalensis* via forward vs. reverse complement (palindromic) alignment [31]. The following settings were used for repeat identification: (1) 90% or greater sequence identity; (2) Hamming distance of 3; and (3) a minimum repeat size of 30 bp.

### 2.5. Interspecific Comparison

The complete chloroplast genomes of *S. baicalensis* and the two other species of *Scutellaria* (*S. insignis* and *S. lateriflora*) from GenBank were compared using mVISTA in Shuffle-LAGAN mode [32]. These chloroplast genome sequences were aligned using MAFFT v7 [33] and adjusted manually where necessary using Se-Al 2.0 [34]. Sliding window analysis was conducted to determine the nucleotide diversity of the chloroplast genome using DnaSP v5.10 software [35]. The step size was set to 200 bp, with an 800-bp window length.

### 2.6. Intraspecific Comparison

The two complete chloroplast sequences of *S. baicalensis* were aligned using MAFFT v7 [33] and adjusted manually where necessary using Se-Al 2.0 [34]. We recorded SNPs (single nucleotide polymorphisms) and indels (insertion/deletions) separately as well as their locations in the chloroplast genome. Substitution sites and genetic distance in the chloroplast genome were calculated using MEGA 6.0 [29]. Based on the aligned sequence matrix, the indel events were checked manually and were further divided into two categories: microsatellite-related indels (SSR-indel) and non-microsatellite-related indels (NR-indel).

### 2.7. Phylogenetic Reconstruction

As the plastid genome is present in high copy numbers in each cell in most plants and shows relatively little variation in gene content and order, the chloroplast genome has been widely used to resolve phylogenetic relationships among plant lineages [18,36]. To further identify and validate the phylogenic relationships of *Scutellaria* with other Lamiaceae species, phylogenetic analysis was conducted using published chloroplast genomes, including 31 species from Lamiaceae. The chloroplast genome sequences of *Erythranthe lutea*, *Rehmannia chingii* and *Lindenbergia philippensis* were employed as out-groups. In this study, we concatenated the 84 chloroplast genes for phylogenetic analyses. The sequences were aligned using MAFFT v7 [33], and the alignment was manually adjusted.

The program JModeltest 2 was employed to find the optimal substitution model [37], using both the Bayesian information criterion and the Akaike information criterion. The general time-reversible model of substitution, incorporating invariant sites and a gamma distribution (GTR + I + G), was among a group of equally best fitting models (found in the 100% confidence interval) and was employed in subsequent analyses. Maximum likelihood (ML) phylogenetic tree analysis was conducted using RAxML v8.0 [38]. Bootstrap support was estimated with 1000 bootstrap replicates.

Bayesian inference (BI) was implemented with MrBayes 3.2.2 [39]. The Markov chain Monte Carlo (MCMC) analysis was run for 2 × 10,000,000 generations. The trees were sampled every 1000 generations, with the first 25% discarded as burn-in. The remaining trees were used to build a 50% majority-rule consensus tree. Analysis was run to completion, and the average standard deviation of the split frequencies was <0.01.

## 3. Results

### 3.1. Genome Size and Features

**Figure 1:**
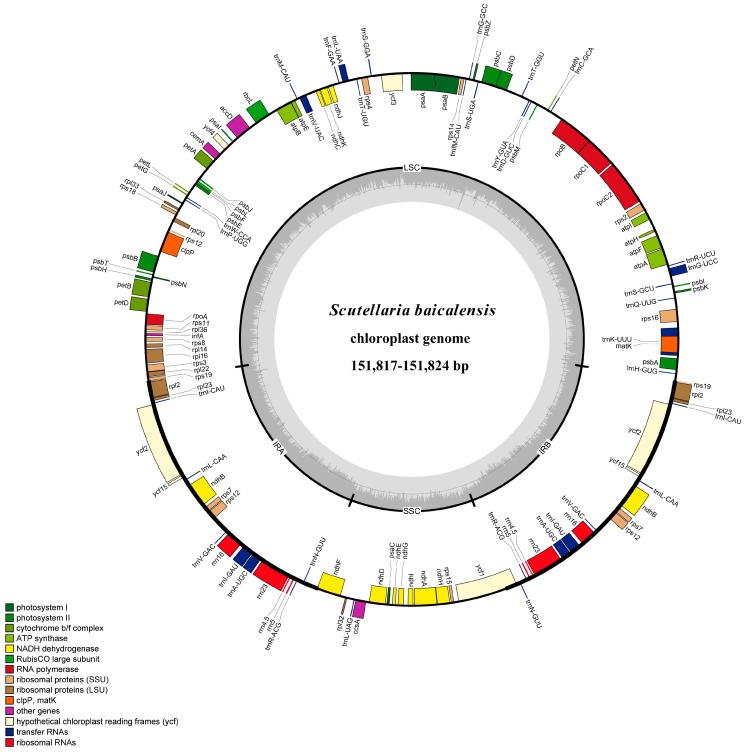
Chloroplast genome map of *Scutellaria baicalensis*. The genes drawn outside of the circle are transcribed clockwise, while those inside the circle are transcribed counterclockwise. Small single copy (SSC), large single copy (LSC), and inverted repeats (IRa, IRb) are indicated.

The total lengths of the sequences from the 2 *S. baicalensis* genotypes were determined to be 151,817 and 151,824 bp, with a circular quadripartite structure similar to that of major angiosperm chloroplast genomes. The chloroplast genomes contained an SSC region of 17,331 or 17,338 bp, and an LSC region of 83,960 or 83,976 bp, separated by 2 copies of an IR of 25,263 or 25,255 bp (Table 1 and Figure 1). The annotated genome sequences have been submitted to GenBank (accession number MF521632 and MF521633).

In the *S. baicalensis* chloroplast genome, 114 unique genes were identified, including 80 protein-coding genes, 30 tRNA genes, and 4 rRNA genes (Figure 1 and Table S1). The LSC region contained 61 protein-coding genes and 22 tRNA genes, whereas the SSC region contained 12 protein-coding genes and 1 tRNA gene. Seven protein-coding genes, 7 tRNA genes and all 4 rRNA genes were duplicated in the IR regions.

In the *S. baicalensis* chloroplast genome, there were 18 intron-containing genes, 16 of which (10 protein-coding and 6 tRNA genes) contained 1 intron, while 2 (*clpP*, and *ycf3*) contained 2 introns (Table S1). The *rps12* gene is a trans-spliced gene whose 5′ end is located in the LSC region, while the duplicated 3′ end is located in IR regions. *trnK-UUU* exhibited the largest intron (2574 bp) and contained the *matK* gene.

The GC content of the *S. baicalensis* chloroplast genome was 38.3%, which is consistent with other reports for Lamiaceae (*Salvia miltiorrhiza*: 38.0% and *Origanum vulgare*: 38.0%) [40,41]. The GC contents of the LSC and SSC regions were 36.3% and 32.7%, respectively, whereas that of the IR region was 43.6%. The high GC content in IR regions was due to the reduced presence of AT nucleotides in the 4 duplicate rRNA genes.

### 3.2. Codon Usage

We further analyzed the codon usage frequency and RSCU in the *S. baicalensis* chloroplast genome. There were 78,747 nt and 26,249 codons in the *S. baicalensis* plastome in total, representing the coding regions of 90 protein-coding genes. Among the codons, leucine (10.62%) and cysteine (1.14%) were the most- and least-abundant amino acids, respectively (Table S2). Codon usage was biased towards A and T at the third codon position, which is similar to the trend observed in a majority of angiosperm plastid genomes.

### 3.3. Repeat and Microsatellites Analysis

**Figure 2:**
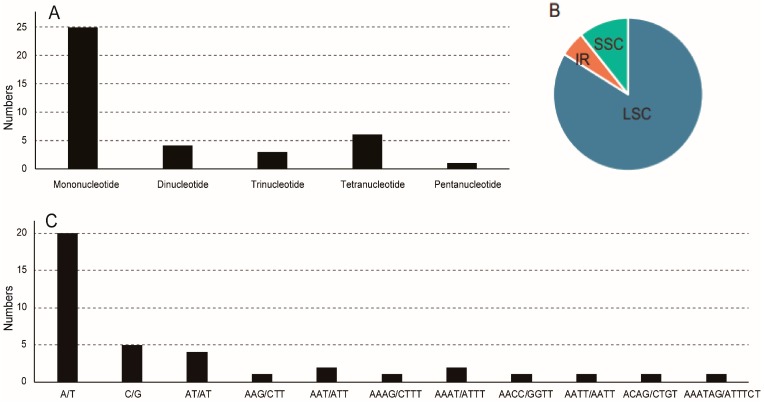
The distribution, type and presence of microsatellites (SSRs) in the chloroplast genome of *Scutellaria. baicalensis*. (**A**) Number of different SSR types; (**B**) Proportion of SSRs in LSC, SSC, and IR regions; (**C**) Number of identified SSR motifs in different repeat class types.

SSRs, also known as microsatellites, are tandem repeated DNA sequences that are generally 1–6 bp in length per unit and are distributed throughout the genome. Using the microsatellite identification tool MISA, 39 SSRs were identified in the *S. baicalensis* chloroplast genome (Figure 2 and Table S3). In the *S. baicalensis* chloroplast genome, 5 compound microsatellites were detected (Figure 2). Among these SSRs, there were 25 homopolymers, 4 dipolymers, 3 tripolymers, 6 tetrapolymers and 1 hexapolymer. Among the 29 homopolymers and dipolymers, only 5 SSRs contained G or C bases. A total of 31 SSR loci were located in LSC regions, 4 in SSC regions, and 4 in IR regions. Most of these SSRs were located in intergenic regions (30 SSRs), while 7 SSRs were located in introns, and only 2 were located in protein-coding genes (*accD* and *ndhD*). We designed primer pairs for the amplification of all SSRs (Table S3).

With the exception of SSRs, repeats with a length ≥30 bp were considered long repeat sequences in the *S. baicalensis* chloroplast genome. A total of 38 long repeat sequences were detected (Table S4), including 16 direct (forward) repeats and 22 inverted (palindrome) repeats. Approximately 80% of these repeats fell exclusively within intergenic regions, whereas 20% were located within or at their border of genes. Most of these repeats exhibited lengths between 30 and 41 bp, while *petN-psbM* possessed the highest number of repeats (11) and exhibited the longest repeats, of 130 bp.

### 3.4. Interspecific Comparison

**Figure 3:**
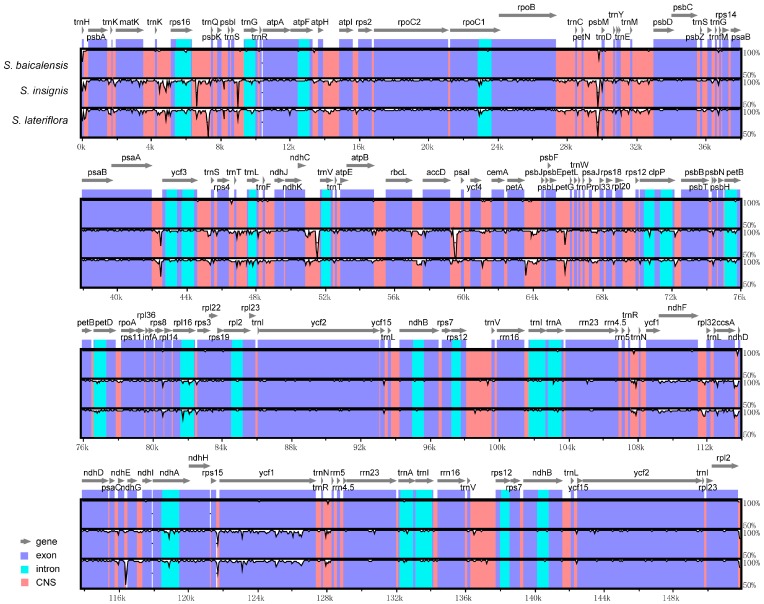
Visualization of the alignment of chloroplast genome sequences of *Scutellaria*. VISTA-based similarity graphical information illustrating the sequence identity of *Scutellaria* with reference *S. baicalensis* chloroplast genomes. Grey arrows above the alignment indicate the orientation of genes. Purple bars represent exons; blue bars represent introns; and pink bars represent non-coding sequences (CNS). A cut-off of 50% identity was used for the plots. The Y-scale axis represents the percent identity within 50–100%. Genome regions are color-coded as protein-coding exons, rRNAs, tRNAs, or conserved CNS.

Interspecific comparisons of sequence identity among the 3 chloroplast genomes were conducted with mVISTA using the annotated *S. baicalensis* sequence as a reference (Figure 3). The mVISTA results showed that the 3 chloroplast genomes were highly conserved; however, the non-coding regions appeared to be more variable globally than the coding regions. The overall sequence divergence estimated based on the p-distance among the 3 genomes was only 0.0078. The pairwise p-distance between 3 species ranged from 0.0041 to 0.0099.

**Figure 4:**
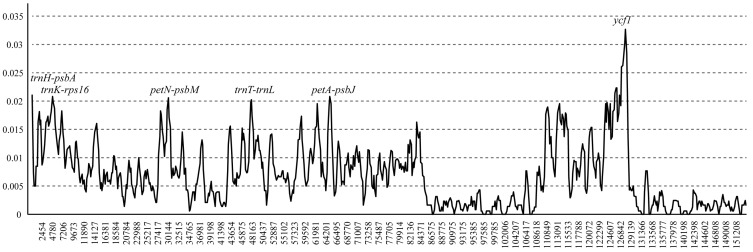
Sliding window analysis of the whole chloroplast genomes of three *Scutellaria* species. Window length: 800 bp; step size: 200 bp. X-axis: position of the midpoint of a window. Y-axis: nucleotide diversity of each window.

Furthermore, sliding window analysis using DnaSP detected highly variable regions in the *Scutellaria* chloroplast genome (Figure 4). The average value of nucleotide diversity (PI) was 0.00775. The IR regions exhibited lower variability than the LSC and SSC regions. There were 6 mutational hotspots that showed remarkably higher PI values (>0.02), including 1 gene region (*ycf1*) and 5 intergenic regions (*trnH-psbA*, *trnK-rps16*, *petN-psbM*, *trnT-trnL*, and *petA-psbJ*) from the chloroplast genomes. These regions may be undergoing more rapid nucleotide substitution at the species level, indicating potential application of molecular markers for phylogenetic analyses and plant identification in *Scutellaria*.

### 3.5. Intraspecific Comparison

The 2 chloroplast genomes from *S. baicalensis* were found to show only a 7 bp difference in length (Table 1). In addition to the total length difference, we assessed SNP and indel variations between the 2 chloroplast genomes of *S. baicalensis* in their entirety. There were 25 SNPs (8 transversions and 17 transitions) identified in the chloroplast genomes (20 in LSCs and 5 in SSCs; Table S5). The most frequently occurring mutations were C/T substitutions (11 times), while A/T and G/A mutations exhibited the lowest frequency (only 1 occurrence of each). Sixteen, 3 and 6 SNPs were located in intergenic, intron and coding regions, respectively. Two SNPs in *rpoC2* and *ndhI* resulted in synonymous changes. *petA-psbJ* contained the highest number of SNPs (four), followed by *trnH-psbA*, *ycf4-cemA* and *ndhF*, each of which displayed 2 SNPs. All of the other 15 regions contained only 1 SNP.

There were 29 indels in the chloroplast genome identified between the 2 *S. baicalensis* genotypes (Table S6), including 21 indels caused by SSR variations (SSR-indels) and 8 non-SSR-related indels (NR-indels). All of the SSR-indels were related to homopolymers (16 A/T types and 5 C/G types) and occurred within non-coding regions. The other 8 indels were located in 8 different regions (seven in intergenic regions and 1 in an intron region). *petN-psbM* exhibited the longest indel, of 26 bp in length.

### 3.6. Phylogenetic Reconstruction of Lamiaceae

Recent advances in high-throughput sequencing have provided large amounts of data, which has improved phylogenetic resolution. The chloroplast genome has been widely employed as an important source of molecular markers in plant systematics. To determine the phylogenetic position of *Scutellaria* in Lamiaceae, 34 complete chloroplast genome sequences were obtained from GenBank (Table S7). For phylogenetic analysis, 84 concatenated coding genes were used, representing the entire chloroplast genome. The sequence alignment data matrix employed for phylogenetic analysis comprised 73,820 nucleotide positions, including 13,900 variable sites (18.83%) and 7968 parsimony-informative sites (10.79%).

**Figure 5:**
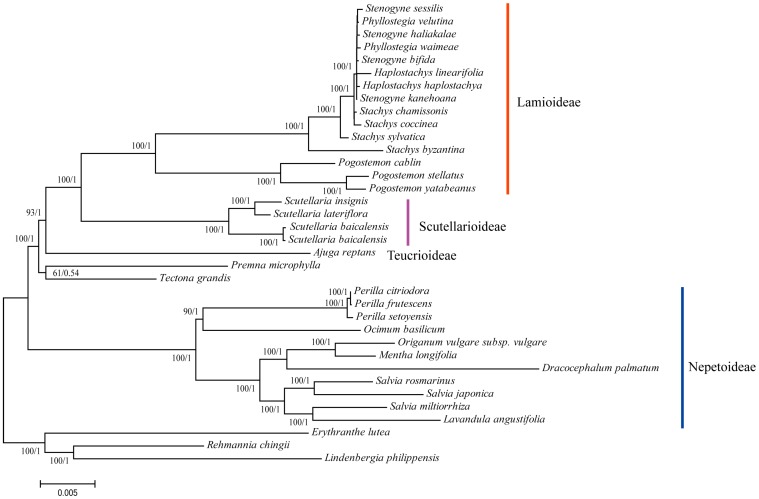
Phylogenetic tree reconstruction of 36 taxa using maximum likelihood and Bayesian inference based on the concatenated sequences of 84 genes. Maximum likelihood (ML) topology, with the ML bootstrap support value/Bayesian posterior probability given at each node.

The ML and BI trees exhibited similar phylogenetic topologies (Figure 5), and the phylogenetic tree formed 4 major clades: Lamioideae, Scutellarioideae, Teucrioideae, and Nepetoideae. The positions of *Premna microphylla* and *Tectona grandis* within Lamiaceae were uncertain. *Scutellaria baicalensis* was the closest sister species of *S. lateriflora* and *S. insignis*, with bootstrap values of 100%. The differing branch lengths within Lamiaceae suggest a heterogeneous evolutionary history between these clades with regard to chloroplast evolution. The phylogenetic positions of these groups are in agreement with the findings of recent studies [42,43].

## 4. Discussion

In this study, we generated the chloroplast genomes of two *S. baicalensis* genotypes, providing important resources for genetic engineering as well as evolutionary and species identification studies. The obtained chloroplast genomes of *S. baicalensis* exhibit the typical angiosperm quadripartite structure, and their gene content and order and GC content were consistent with those of most other members of Lamiaceae [40,41,43].

Highly variable markers in chloroplast genomes between different species at the genus level have provided abundant informative loci for systematic plant and DNA barcoding research [16,21]. We compared interspecific chloroplast diversity in *Scutellaria*. We found more variation, with an average nucleotide diversity value of 0.0075, among the three *Scutellaria* species compared with *Machilus* [20], *Quercus* [44], and *Lagerstroemia* [21]. *Scutellaria* species are perennial herbs with short life histories that usually evolve relatively quickly and exhibit high substitution rates [45,46]. In this study, we identified six highly variable regions (candidate DNA barcodes), including *trnH-psbA*, *trnK-rps16*, *petN-psbM*, *trnT-trnL*, *petA-psbJ*, and *ycf1*. *TrnH-psbA* loci are highly variable in most plant groups, and inversions or mononucleotide repeats occur within these loci, which may result in incorrect alignments or sequencing difficulties [47,48]. *trnK-rps16*, *petN-psbM*, *trnT-trnL*, and *petA-psbJ* have been used in previous phylogenetic studies. The most variable of the identified loci was *ycf1*, which is the second-largest gene in the chloroplast genome, encoding a protein of approximately 1800 amino acids [17]. The variance of *ycf1* was about twice that of the other four loci in *Scutellaria* (Figure 4). *ycf1* may represent the best barcode specific to *Scutellaria* that is currently available.

Within the *S. baicalensis* group, we were able to retrieve SNPs, SSRs, and indels. A total of 25 SNPs was identified between the chloroplast genomes of the two *S. baicalensis* genotypes (Table S5). Mononucleotide SSRs (particularly of As and Ts) are the most abundant and widely studied microsatellites in the non-coding regions of the chloroplast genome [49]. Few repeats of more than two nucleotides have previously been reported in Lamiaceae [50]. We classified the identified indels into two types: SSR-indels and NR-indels. Several molecular processes are known to generate indels. Slipped strand mispairing (SSM), which adds or subtracts short repeat sequences, has been suggested as the likely mechanism responsible for most SSR-indels [51,52]. Larger indels are often associated with secondary structure formations and/or localized or extra-regional intramolecular recombination [51]. There were 21 SSR-indels identified in the two genotypes, indicating that these SSRs were polymorphic (Table S6). Indels in the chloroplast genome have been shown to be extremely useful for resolving phylogenetic relationships between closely related taxa and therefore increase the power of intraspecific studies [53,54].

## 5. Conclusions

The chloroplast genomes obtained from two *S. baicalensis* genotypes were compared with two other *Scutellaria* chloroplast genomes, allowing us to compare genomic diversity at different taxonomical levels and to develop intraspecific and interspecific molecular markers. These resources can now be employed to explore the variation of *Scutellaria* populations and for further evolutionary, phylogenetic, barcoding and genetic engineering studies, in addition to effective exploration and conservation of *S. baicalensis*.

## Figures and Tables

**Table 1:** Summary of the characteristics of *Scutellaria baicalensis* chloroplast genome.

Genotype	BOP028265	BOP025266
Total cpDNA size (bp)	151,824	151,817
LSC size (bp)	83,976	83,960
IR size (bp)	25,255	25,263
SSC size (bp)	17,338	17,331
Number of genes	114	114
Number of different protein-coding genes	80	80
Number of different tRNA genes	30	30
Number of different rRNA genes	4	4
Number of different duplicated genes	17	17
GC content	38.3%	38.3%
GC content of LSC	36.3%	36.3%
GC content of SSC	32.7%	32.7%
GC content of IR	43.6%	43.6%

cpDNA: chloroplast DNA; LSC: large single-copy; IR: inverted repeat; SSC: small single-copy.

## Supplementary Materials

The following are available online at www.mdpi.com/2073-4425/8/9/227/s1. Table S1: List of genes found in the *S. baicalensis* chloroplast genome, Table S2: Codon usage and RSCU of *S. baicalensis* chloroplast genome, Table S3: Primer pairs for SSRs of *S. baicalensis*, Table S4: Distribution and location of tandem repeats in *S. baicalensis* chloroplast genome, Table S5: The nucleotide substitution patterns present in the *S. baicalensis* chloroplast genomes, Table S6: Indel mutation events in the chloroplast genomes of *S. baicalensis*, Table S7: List of chloroplast genomes sequences employed for phylogenetic analysis.
